# New insights into the effects of microbiome and its derived metabolites on targeted immunotherapy

**DOI:** 10.1186/s43046-025-00330-x

**Published:** 2025-11-16

**Authors:** Maged Tharwat Elghannam, Moataz Hassan Hassanien, Yosry Abdel Rahman Ameen, Emad Abdelwahab Turky, Gamal Mohammed ELattar, Ahmed Aly ELRay, Mohammed Darwish ELTalkawy

**Affiliations:** Hepatogastroenterology deparment, Theodor Bilharz Research Institute, Giza, Egypt

**Keywords:** Gut microbiota, Bacterial metabolites, Targeted therapy, Drug-resistance

## Abstract

The significance of gut bacteria and their byproducts is gaining greater recognition, especially in the realm of immunotherapy. An imbalance in gut bacteria or their byproducts is intricately linked to the onset, progression, and treatment of cancer. Metabolites derived from gut microbiota, including short-chain fatty acids (SCFAs), secondary bile acids (SBAs), indole derivatives, and trimethylamine oxide (TMAO), engage with cellular targets to initiate intracellular signaling pathways. These signals are conveyed to the cell, influencing its growth. Targeted therapies encompass a complex and ever-evolving area that is crucial in cancer management. Nonetheless, it is vital to recognize that targeted therapy encounters a multitude of challenges. Factors influencing the success of targeted therapy include drug resistance resulting from prolonged use, side effects, and variations in genetic mutations, tumor diversity, and the complex nature of the tumor microenvironment. Recently, we have deepened our understanding of the relationship between the gut microbiome and anticancer targeted therapies. This is one face of the molecular pathologic epidemiology. This prompts us to investigate promising treatment strategies linked to these gut bacteria and their metabolites, thereby unlocking new possibilities for targeted anticancer therapies.

## Introduction

The gastrointestinal microbiota is a complex community of bacteria, archaea, fungi, and viruses present in the host’s digestive tract. The genetic material of the microbiota in addition to the surrounding environmental conditions are referred to as the microbiome [1, 2]. Microbiota has many essential functions that keep sustaining the homeostasis of the digestive system. Microbiota preserve the intestinal barrier with a consequence of proper digestion and absorption of nutrients. In addition, microbiota regulate the function of the immune system and provide defense against pathogenic invasions . Various factors can offend the homeostatic status of the gut bacteria. These factors include drugs, diet, and genetic susceptibility [3, 4].

Gastric microbiota include 5 dominant phyla, namely, *Anaplasma*, Firmicutes, *Clostridium*, *Actinobacteria*, and *Aspergillus* (including *H. pylori*). However, the dominant phyla attached to the gastric mucosa include *Firmicutes* and *Aspergillus* phyla. In contrast, the gastric lumen is most commonly colonized by the Firmicutes, *Clostridium*, and Actinobacteria phyla [5].

Gastrointestinal microbes and their metabolites contribute significantly to the process of malignant transformation in a multifaceted manner. They aid in tumor metastasis, influence the efficacy of immunotherapy, and are responsible for chemotherapy resistance [6]. Although the microbiota may be present in areas far away from the gut, as in the oral cavity, lung, or skin, they can influence malignant biology and transformation whether initiation or progression in interaction with gut microbiota [7, 8].

Targeted therapies constitute a sophisticated and ever-evolving field that is essential in cancer therapy. These therapies are extensively applied across a range of cancer types, such as solid and hematologic tumors [9, 10]. The particular molecular targets include the vascular endothelial growth factor receptor (VEGFR) [11], epidermal growth factor receptor (EGFR) [12], human epidermal growth factor receptor 2 (HER2) [13], and insulin-like growth factor 1 receptor (IGF-1R) [14]. These targets are vital molecules that influence cancer cell survival, proliferation, and metastasis. Targeted therapies can, with a high degree of accuracy, destroy cancer cells with considerable benefits over conventional chemotherapy, such as the capacity to customize treatment plans according to the patient’s distinct genetic profile and disease condition. Such personalization increases specificity, reduces harm to adjacent healthy cells, and thereby lessens side effects while enhancing patient outcomes. However, opposing force that may reduce drug effectiveness include prolonged use that lead to drug resistance, side effects, drug pressure causing genetic mutations, the tumor heterogenic cellular structure, and the intricate nature of the surrounding tumor environment. Furthermore, individual pharmacokinetics and pharmacodynamics in each individual patient that affect drug absorption, distribution, and metabolism add to the discrepancies in treatment results. Although the FDA approved many drugs such as rituximab, cetuximab, and bevacizumab [15], still, it is difficult to anticipate how high the competency of the targeted therapy is [16]. This review aims to illustrate how the microbiome and its derived metabolites can affect the response to immune target therapy and explain differences in disease outcomes and response to treatment.

## Molecular pathological epidemiology (MPE)

Pathology and epidemiology have been integrated into the single, unified field of molecular pathological epidemiology (MPE). Most human diseases, and essentially all cancers, are biologically different from one patient to the next.

**Fig. 1:**
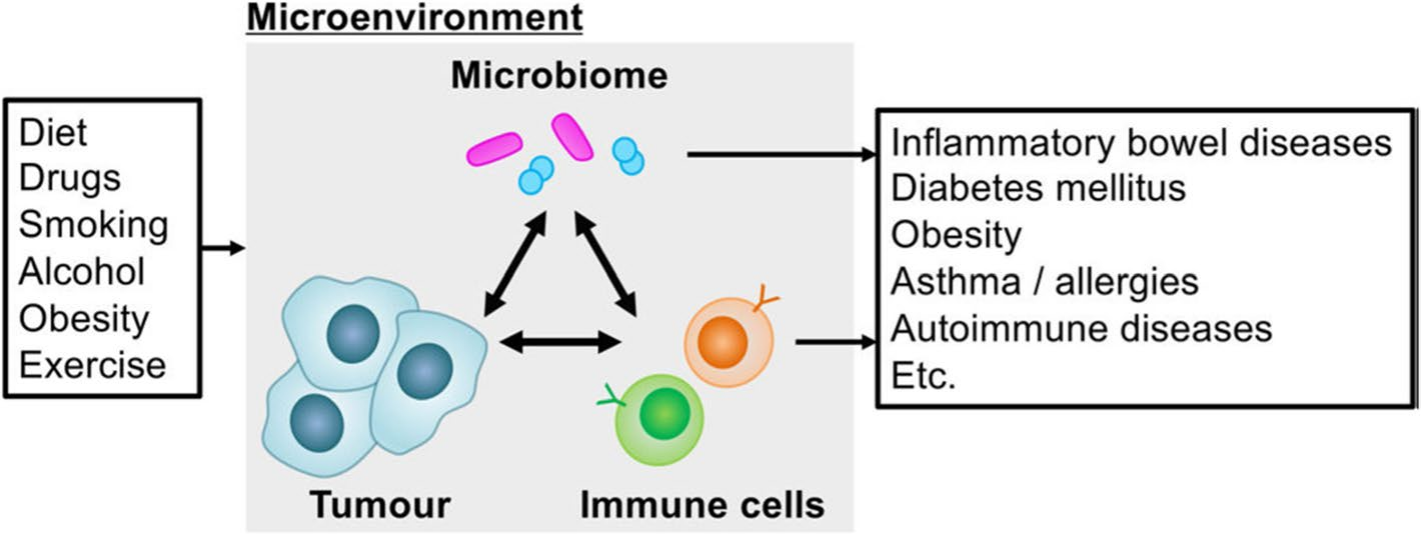
Interactions of the microbiome, host immune system, and tumor in the microenvironment. The imbalance of microbial communities may cause neoplasms as well as non-neoplastic diseases. J Pathol. 2019 April; 247(5): 615–628. 10.1002/path.5236

The primary aim of MPE is to uncover an interactive relationship between a specific environmental exposure and disease subtypes in determining disease incidence and mortality [17]. The MPE approach can be used to study any disease. In addition to cancer research, MPE approach can be applied to non-neoplastic diseases such as cardiovascular diseases, obesity, diabetes mellitus, drug toxicity, and immunity-related and infectious diseases [18] (Fig. 1) [19].

**Fig. 2:**
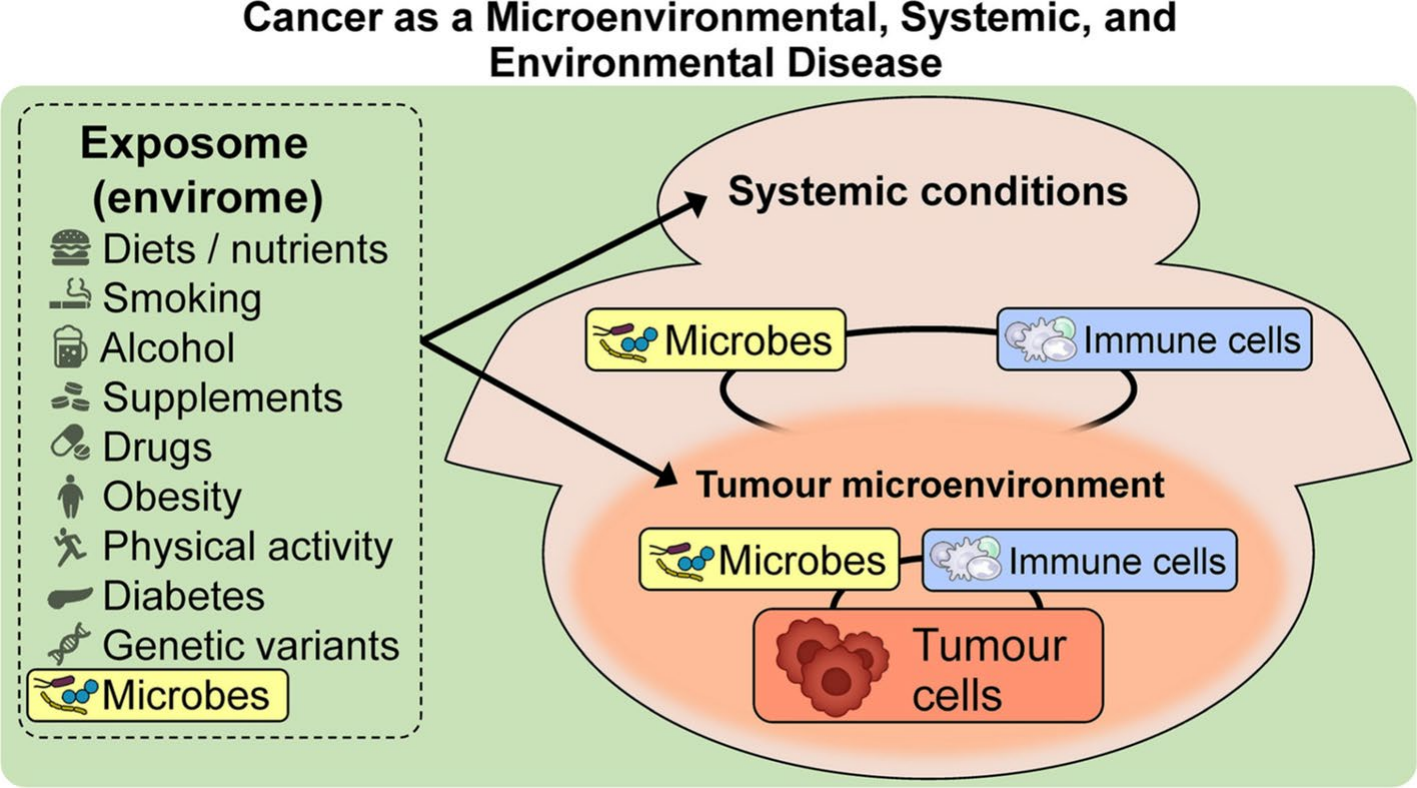
Cancer as a microenvironmental, systemic, and environmental disease. Tumor generates its intrinsic microenvironment, where tumor cells interact with immune cells, microbes, and other cells as well as non-cellular components. Gut 2022; 71:2107–2122

Cancer can be regarded as a microenvironmental, systemic, and environmental disease. The exposome (i.e., the totality of exposures), which encompasses diets, supplements, smoking, alcohol, other lifestyle factors, medications, etc., likely alters the microbiome (inclusive of bacteria, viruses, archaea, fungi, parasites, etc.) and immune system in various body sites and influences tumor phenotypes (Fig. 2) [20].

These factors may vary in each person differentially, which can explain differences in disease outcomes and responses to treatment. The evolving field of MPE can be a core field in the era of big-data health science and precision medicine.

## Intestinal bacteria-derived metabolites and the intracellular signaling targets

Gut bacteria metabolites include short-chain fatty acids (SCFAs), secondary bile acids (SBAs), indole derivatives, and trimethylamine-N-oxide (TMAO). There are research data that strongly link the microbiota metabolites to the carcinogenesis process and the competency of the anti-cancer therapy [21].

1. LPS (Lipopolysaccharide): It is a cell wall component of the gram-negative bacteria [22]. Lipid A component is the main toxic element [23]. Upon the death of bacteria, LPS is liberated into the intestinal environment, then to the bloodstream [24]. There is a significant link between LPS derived from gut bacteria and molecules related to anti-cancer targeted therapies. Zhu et al. [25] demonstrated that LPS activates Toll-like receptor 4 (TLR4)/NF-κB pathway in colonic cancer (CRC) cells. Consequently, via the increase in VEGFR-3 transcription, CRC increases its invasion capability. In hepatocellular carcinoma (HCC) cells, LPS stimulates the elevation of EREG expression in tumor tissues, and through IL-8 signaling, there is enhancement of tumor angiogenesis which promotes migration and invasion [26]. LPS activates the mTOR/NF-κB pathway by enhancing TLR4/MyD88/MAPK signaling [27]. Additionally, LPS stimulates the mTOR/STAT3 pathway [28]. Consequently, both receptor tyrosine kinases (RTKs) and the PI3K/AKT/mTOR signaling pathway have been enhanced, hindering antitumor therapies’ effect that target this signaling pathway.
2. Secondary bile acids: They include deoxycholic acid (DCA), lithocholic acid (LCA), and ursodeoxycholic acid (UDCA) [29]. They are essential for lipid digestion and absorption, and activate specific receptors such as FXR and GPCRs, which significantly impact energy metabolism and immune modulation [29, 30]. Gut microbiota has the ability to synthesis secondary bile acids. However, *Eubacterium lentum* converts bile acids especially to UDCA. Additionally, other gut bacteria like *Bacteroides*, *Faecalibacterium*, and *Ruminococcus* also produce secondary bile acids, influencing specific targets and exacerbate several diseases [31]. Secondary bile acids have the ability to influence RTKs expression [32, 33]. In rat HCC cells, DCA induce phosphorylation of the EGFR leading to the upregulation of mucin 2 (MUC2) and facilitation of cancer invasion [34]. A high-fat diet (HFD) increases levels of the microbial metabolite DCA, which activates VEGFR2 signaling and contributes to high rate of colon cancer. Conversely, DCA may also directly target HER2, potentially exerting an anti-cancer effect. DCA could inhibit antitumor therapies aimed at RTKs and HER2. Apoptosis, whatever its cause, can be aborted by UDCA denoting a probable role in cancer prevention [35]. UDCA not only inhibits the activation of the EGFR/ERK pathway, which reduces the proliferation of malignant cells, but it also suppresses the expression of PI3K and AKT, further supporting its anti-tumor properties in these cells [36]. Additionally, UDCA can block IGF-1-induced activation of AKT and ERK, which are crucial pathways for regulating the growth of cholangiocarcinoma cells [37]. In the context of hepatocellular carcinoma (HCC), UDCA enhance the sorafenib-induced downregulation of phospho-STAT3, thereby amplifying its inhibitory effect on tumor growth [38]. Overall, UDCA may improve anti-tumor therapies that target RTKs, STAT, and the PI3K/AKT signaling pathway (Fig. 3).

**Fig. 3:**
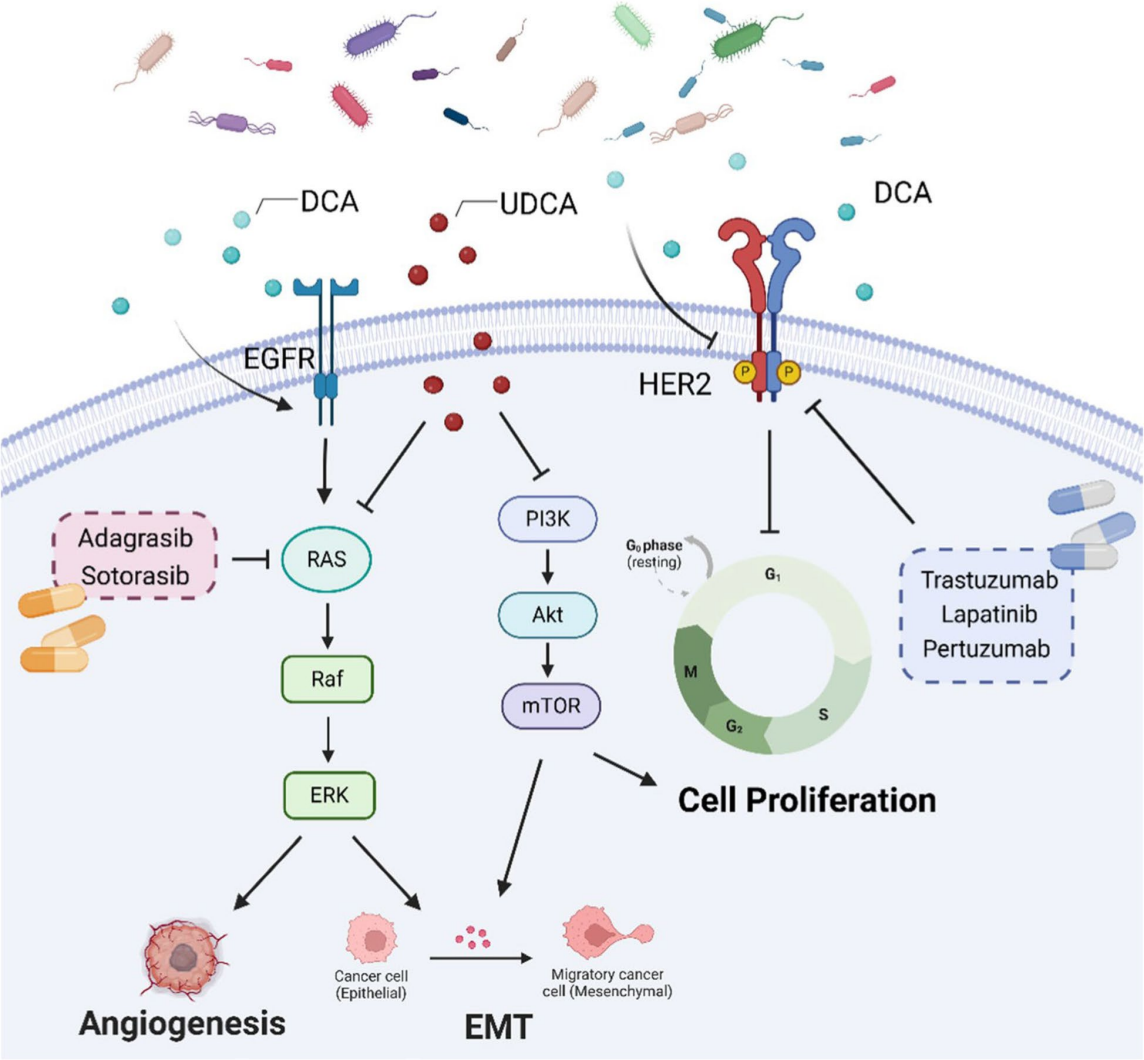
Secondary bile acids and their targets. DCA could inhibit antitumor therapies aimed at RTKs and HER2. UDCA inhibits both the activation of the EGFR/ERK pathway, which reduces the proliferation of malignant cells, and suppresses the expression of PI3K and AKT, further supporting its anti-tumor properties in these cells. Overall, UDCA may improve anti-tumor therapies that target RTKs, STAT, and the PI3K/AKT signaling pathway. Published by He et al. in Mol Med 31, 58 (2025)
3. Short-chain fatty acids (SCFA): Gut microbiota ferment the indigestible dietary fibers to produce SCFAs, such as acetic acid derivation, propionate, and butyrate [39]. The abundance of SCFA-producing microbiota in the intestine, along with dietary fiber consumption, influences the concentration of SCFAs in both stool and plasma [39, 40]. Microbes that produce butyrate primarily consist of bacteria from the families Ruminalococcaceae and Lachnospiraceae, along with species such as Anaerobutyricum hallii and Anaerostipes spp. [41–43]. In differentiate, acetic acid derivatives and propionate are predominantly produced by Bifidobacterium and mucin-degrading microorganisms, including Akkermansia muciniphila [44]. Butyrate is accepted to play a noteworthy part within the event [45], advancement, and treatment of tumors. Butyrate serves as a crucial energy source for colonic epithelial cells and also regulates gene expression by inhibiting histone deacetylase (HDAC) [46, 47], subsequently influencing cell expansion, differentiation, and apoptosis. Additionally, butyrate serves as a local signaling molecule within the cellular digestion system [48], influencing tumor cells and playing a crucial role in modulating the tumor microenvironment and immune response [49, 50]. It has been demonstrated that butyrate downregulates VEGF expression in a dose-dependent manner [51, 52]. By reducing VEGF expression and angiogenesis, butyrate effectively decreases tumor metastasis [53]. Furthermore, butyrate inhibits the VEGF and JAK2/STAT3 signaling pathways, thereby exerting anti-tumor effects. Dariushnejad et al. [54] discovered that sodium butyrate, at a specific concentration, enhanced the expression of VEGFR2 and several proteins associated with angiogenesis, including NOx, AKT, ERK1/2, and VEGF-A. According to the tumor type, butyrate has distinctive impact. SCFAs have a regulatory role in the expression of human insulin-like growth factor-1 (IGF-1), a pivotal hormone for substantial development, however a negative role in prostate cancer. Matsushita et al. [55] illustrated that SCFA-induced IGF-1 generation from intestine microbes impacts prostate cancer development by activating neighborhood prostate MAPK and PI3K signaling pathways, subsequently proposing the presence of a intestine microbiota-IGF-1-prostate hub. Furthermore, Yan et al. discovered that short-chain fatty acids (SCFAs) elevate systemic levels of insulin-like growth factor 1 (IGF-1), thereby playing a role in bone structure and development [56]. Generally, SCFAs had been proposed to impact illness progression by directing the expression of VEGF.
4. Trimethylamine oxidase (TMAO): TMAO is a metabolite produced by intestinal bacteria and the liver [57]. It is formed by intestinal flora from dietary components such as choline, lecithin, and carnitine, which are primarily derived from sources like meat, fish, and eggs. TMA is absorbed in the intestines and carried through the portal circulation to the liver, where it is converted into TMAO [58]. TMAO serves numerous natural capacities, such as cholesterol metabolism and atherosclerosis regulation, in addition to a potential role in expanded risk of cardiovascular illness [59]. TMAO is included within the control of VEGF expression [60, 61]. Moreover, TMAO influences tumor expansion by means of other targets as in activation of ILK/AKT/mTOR signaling pathway [62]. Through the activation of the NF-κB signaling pathway in endothelial cells and vascular smooth muscle cells (VSMCs), TMAO enhances the expression of cytokines and adhesion molecules. This mechanism can participate in cases of chronic illness such as hypertension, atherosclerosis, thrombosis, and chronic kidney diseases [63]. TMAO enhances the protein kinase R-like endoplasmic reticulum kinase (PERK)/Akt/mTOR signaling pathway [64]. It exerts a harmful influence in cancer by targeting the VEGF, PI3K, NF-κB, and PERK/Akt/mTOR pathways.
5. Indole: It affects gastrointestinal motility causing intermittent bowel habits whether diarrhea or constipation [65, 66]. Gut bacteria partially metabolize dietary tryptophan into indole and its derivatives, which include ligands for the aryl hydrocarbon receptor (AHR), such as indole, indole aldehyde (IAld), and indole acrylic acid [67]. These indole derivatives influence cancer development and progression through the activation of AhR [68–70]. Furthermore, they impact disease mechanisms by targeting the PI3K/AKT pathway and STAT3.
6. Others: Certain metabolites produced by gut bacteria can activate receptors such as NF-κB, PI3K/AKT, and ErbB2/ErbB3, which may play a role in the onset of cancers, including colon cancer. Metabolites from F. nucleatum and Enterotoxigenic Bacteroides fragilis (ETBF) have been found to be prevalent in the intestines of colorectal cancer patients, facilitating the activation of the STAT3 and NF-κB signaling pathways [71]. Conversely, metabolites from lactobacillus casei have been shown to inhibit the expression of ErbB-2 and ErbB-3 genes, thereby reducing tumor proliferation associated with these receptors [72]. Additionally, the production of gamma-aminobutyric acid (GABA) by gut microbiota can modulate VEGF expression, thus affecting tumor progression [73, 74] Fig. 4.

**Fig. 4:**
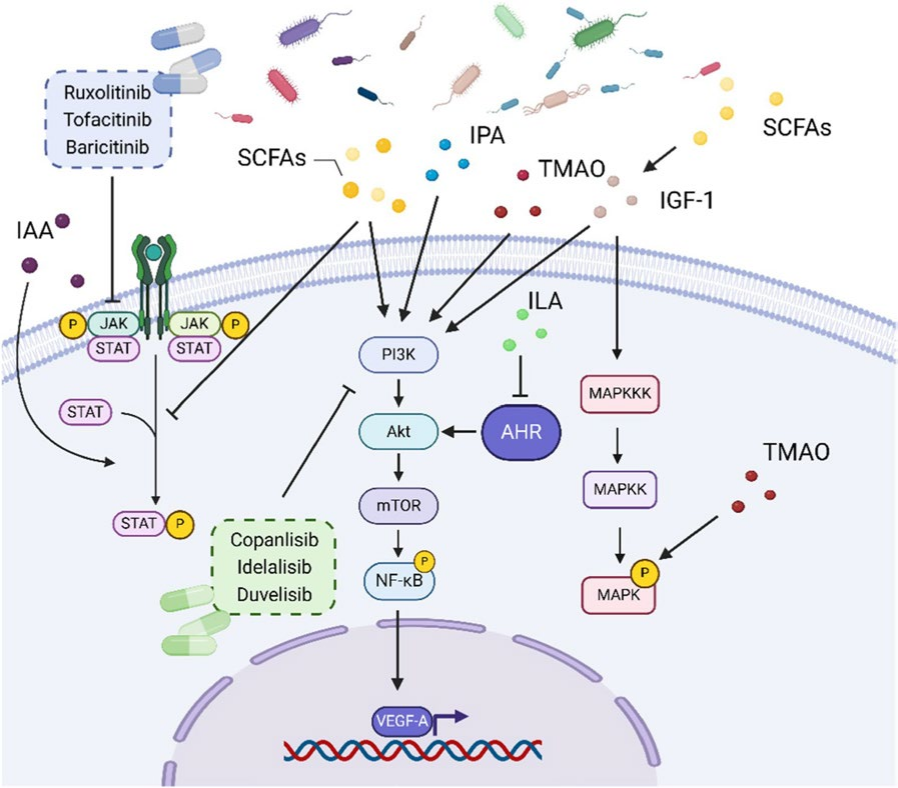
SCFAs, TMAO, and indole derivatives with various targets. Butyrate downregulates VEGF expression and inhibits the JAK2/STAT3 signaling pathways, thereby exerting anti-tumor effects. Indole derivatives impact disease mechanisms by targeting the PI3K/AKT pathway and STAT3. SCFAs elevate systemic levels of insulin-like growth factor 1 (IGF-1). TMAO influences tumor expansion by means of other targets as in the activation of the ILK/AKT/mTOR signaling pathway. Through the activation of the NF-κB signaling pathway, TMAO enhances the expression of cytokines and adhesion molecules. Published by He et al. in Mol Med 31, 58 (2025)

## Inter-relation between gut microbiota, metabolites, and target therapy

By modulating the critical signaling pathways associated with tumorigenesis, intestinal bacteria and their metabolites may improve the effectiveness of targeted therapies. This enhancement can occur through various mechanisms:

### I. Effects of targeted anticancer therapy on gut microbiota

Diarrhea, a common side effect of targeted therapies, is closely associated with dysbiosis of gut bacteria. In patients with metastatic colorectal cancer receiving first-line treatment with anti-VEGF agents (bevacizumab) or anti-EGFR agents (cetuximab), the levels of *Klebsiella*, *Lactobacillus*, *Bifidobacterium*, and *Veillonella* species were significantly elevated in the progressive disease (PD) group compared to the partial response (PR) group. In contrast, *F. nucleatum* and *Prevotella* species were found to be significantly more prevalent in the PR group than in the PD group [75]. Among patients treated with VEGF-tyrosine kinase inhibitors (TKIs), those experiencing diarrhea showed increased levels of Bacteroides spp. and decreased levels of *Prevotella* spp. [76]. Furthermore, in patients with metastatic renal cell carcinoma undergoing treatment with sunitinib, those with diarrhea demonstrated a significantly higher abundance of Bacteroides and a lower presence of butyrate-producing bacteria compared to those without diarrhea [77].

### II. Influence of gut microbiota on targeted anticancer therapy

Growth factors attach to their designated receptors, initiating intracellular signals that are conveyed to cells through a series of signaling pathways, thereby influencing their growth and differentiation. There are two primary intracellular pathways: the PI3K/AKT/mTOR signaling pathway and the signal transducer and activator of transcription (STAT) family. The STAT family functions downstream of the PI3K/AKT/mTOR, Ras/MAPK, and JAK pathways. Tofacitinib inhibits the activity of JAK enzymes, which decreases the phosphorylation of STAT proteins, thus disrupting signaling pathways and contributing to the deceleration of tumor progression while enhancing patient prognosis [78].

### III. Influence of gut microbiota on targeted drug resistance

For targeted therapies to be effective, it is essential that the medicinal compounds are retained within the cell and successfully avoid being expelled by efflux transporter proteins [79]. The intestinal microbiota and their metabolites can affect the ATP-binding cassette (ABC) transporter family, including P-glycoprotein (P-gp) and MDR1 [80, 81], which may contribute to the development of resistance to targeted medications in humans. A nanoparticle model has been developed [82]. The combination of butyrate and bile acids has been shown to enhance the expression of P-gp by either promoting the activity of transcription factors that stimulate P-gp transcription or inhibiting those that suppress it [83]. This illustrates how intestinal bacteria and their metabolites can lead to resistance against targeted therapies.

Even with new discoveries, challenges remain. Analyzing microbes in patient samples is technically hard. Results differ between groups of people. Studies often have too few participants. There is a disconnect between studying microbes and looking at health patterns. We need to test animal findings in actual human tumors. Large prospective studies in people are also necessary. Few researchers have broad skills. Early cancers in the food pipe and airways suggest the gut’s role.

## Conclusion

The gut microbiome and its metabolites are crucial in cancer treatment through various mechanisms. Alterations in gut flora can lead to treatment-related side effects, potentially affecting patient responses and the development of drug resistance. Therefore, understanding changes in gut microbiota may reveal new strategies for enhancing cancer treatment outcomes, reducing side effects, and addressing drug resistance. The gut microbiome and its metabolites interact with several cellular proteins, such as VEGFR, EGFR, and HER2. They influence numerous intracellular signaling pathways associated with cancer treatment, either directly or indirectly, thereby affecting cancer growth, metastasis, and therapeutic responses. Additionally, gut bacteria may impact drug pharmacokinetics and pharmacodynamics, subsequently influencing treatment efficacy by modulating the expression of drug transporter proteins.

## Data Availability

All data are cited inside the manuscript.

## Abbreviations

SCFA: Short-chain fatty acids
SBA: Secondary bile acids
MPE: Molecular pathologic epidemiology
TMAO: Trimethylamine-N-oxide
VEGFR: Vascular endothelial growth factor receptor
EGFR: Epidermal growth factor receptor
HER2: Human epidermal receptor 2
IGF-1R: Insulin growth factor 1 receptor
CRC: Colorectal carcinoma
HCC: Hepatocellular carcinoma
NF-κB: Nuclear factor Kappa B
TLR4: Toll like receptor 4
LPS: Lipopolysaccharide
RTKs: Receptor tyrosine kinases
HDAC: Histone deacetylase
PI3K: Phosphatidylinositol 3-kinase
PKB: Protein kinase B
mTOR: Mechanistic target of rapamycin
STAT: Signal transducer and activator of transcription
PERK: Protein kinase R-like endoplasmic reticulum kinase
